# Crystal structure of 3-amino-1-propyl­pyridinium bromide

**DOI:** 10.1107/S1600536814025665

**Published:** 2014-11-29

**Authors:** P. Venkatesan, V. Rajakannan, S. Thamotharan

**Affiliations:** a School of Chemistry, Bharathidasan University, Tiruchirappalli 620 024, India; b Centre of Advanced Study in Crystallography and Biophysics, University of Madras, Chennai 600 025, India; c Department of Bioinformatics, School of Chemical and Biotechnology, SASTRA University, Thanjavur 613 401, India

**Keywords:** crystal structure, mol­ecular salt, pyridinium salt, N—H⋯Br hydrogen bonds, C—H⋯Br hydrogen bonds

## Abstract

The title mol­ecular salt, C_8_H_13_N_2_^+^·Br^−^, crystallizes with two independent 3-amino­pyridinium cations and two bromide anions in the asymmetric unit (*Z*′ = 2). In the pyridine ring, the N atom is alkyl­ated by a propyl group. The dihedral angle between the mean planes of the pyridinium ring and the propyl group is 84.84 (2)° in cation *A*, whereas the corresponding angle is 89.23 (2)° in cation *B*. In the crystal, the anions and cations are linked *via* N—H⋯Br and C—H⋯Br hydrogen bonds, forming chains propagating along [100].

## Abstract 2

The title mol­ecular salt crystallizes with two independent 3-amino­pyridinium cations and two bromide anions in the asymmetric unit. In the crystal, the anions and cations are linked *via* N—H⋯Br and C—H⋯Br hydrogen bonds, forming chains propagating along [100].

## Chemical context   

Amino­pyridinium and 1-alkyl-amino­pyridinium salts display a wide range of anti­microbial activity (Sundararaman *et al.*, 2013 ▶; Ilangovan *et al.*, 2012 ▶). They have found many applications such as surfactants (Gama *et al.*, 1981 ▶), ionic liquids (Muldoon *et al.*, 2010 ▶; Petkovic *et al.*, 2011 ▶), liquid-crystal display mediums (Ezaki & Kokeguchi, 2006 ▶), ionic crystals for second-order non-linear optics (Anwar *et al.*, 2001 ▶), phase-transfer catalysts in organic transformations (Kupetis *et al.*, 2002 ▶) and additives for protein refolding processes (Yamamoto *et al.*, 2011 ▶). In addition, the amino group in the pyridinium ring participates through hydrogen bonds with wool proteins (Zhao & Sun, 2007 ▶; Calas *et al.*, 2007 ▶).
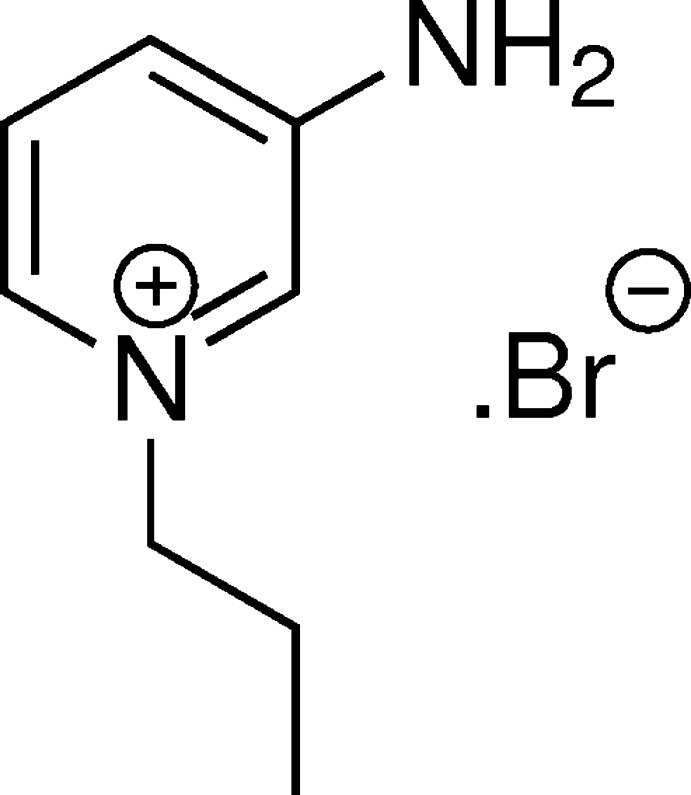


## Structural commentary   

**Figure 1:**
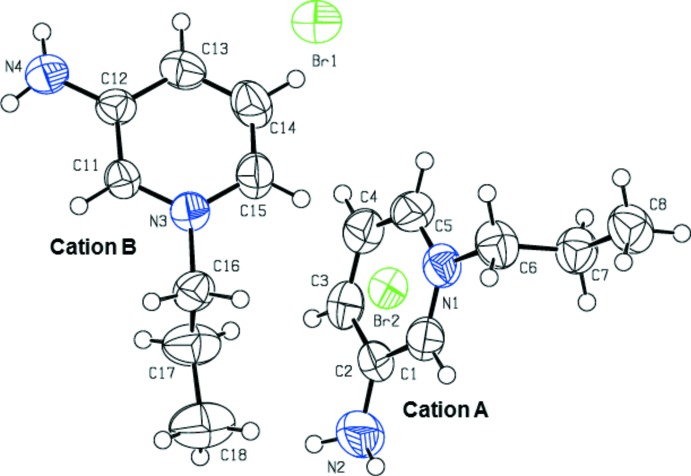
The mol­ecular structure of the title salt, showing the atom labelling. Displacement ellipsoids are drawn at the 50% probability level.

**Figure 2:**
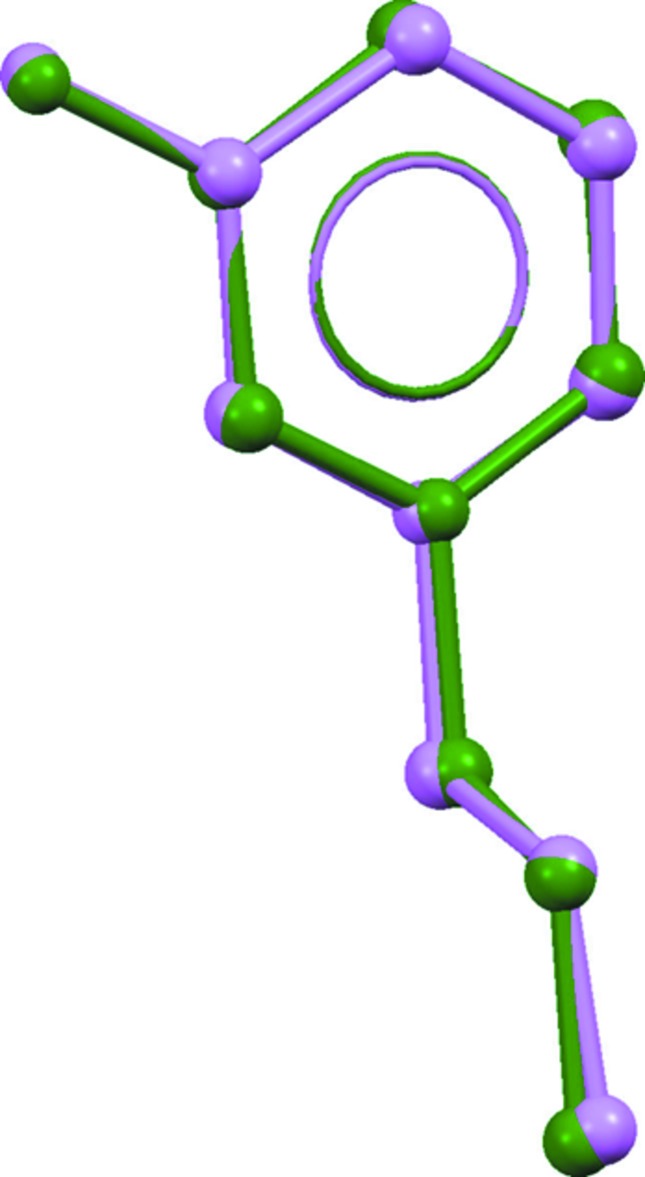
Structural superimposition of the non-H atoms of the pyridinium cations (green: cation *A*; violet: cation *B*).

The asymmetric unit of the title salt, consists of two 3-amino­pyrdinium cations and two bromide anions, as shown in Fig. 1 ▶. The geometrical parameters of the cation moiety are comparable with those of a related structure, 3-amino-1-(4-nitro­benz­yl)pyridinium bromide (Sundar *et al.*, 2006 ▶). The mol­ecular structure of the two cations are very similar with weighted and unit-weight r.m.s. fits of 0.089 and 0.081 Å, respectively, for ten fitted atoms (Fig. 2 ▶). The dihedral angle between the mean planes of the pyridinium ring (N2/C1–C5) and the propyl group (N1/C6–C8) is 84.84 (2)° in cation *A*, whereas the corresponding angle is 89.23 (2)° in cation *B*.

## Supra­molecular features   

**Figure 3:**
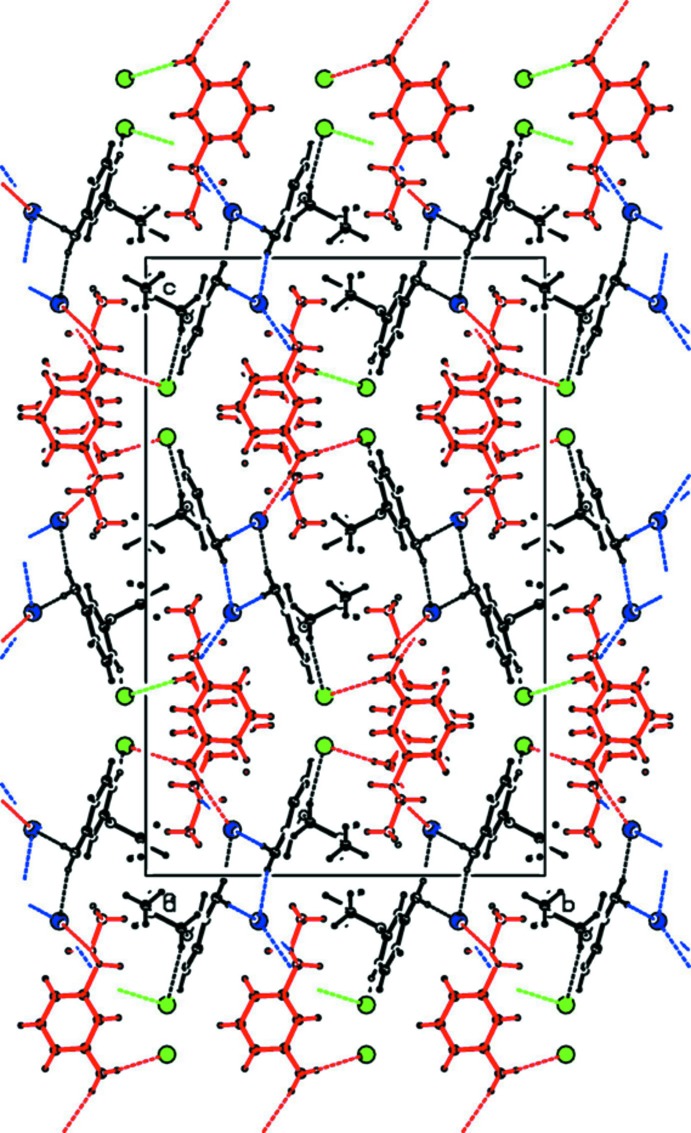
The crystal packing of the title salt projected onto the *bc* plane. The N—H⋯Br and C—H⋯Br hydrogen bonds are shown as dashed lines (see Table 1 ▶ for details).

**Figure 4:**
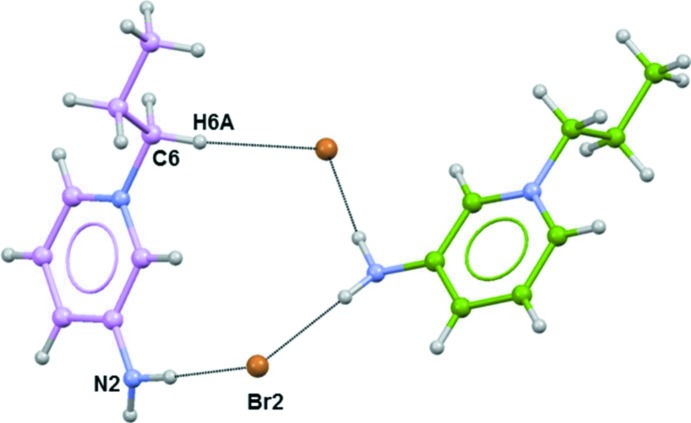
Part of the crystal structure of the title salt, showing the formation of an 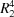
(12) ring motif (see Table 1 ▶ for details; only the inter­acting atoms are labelled).

**Figure 5:**
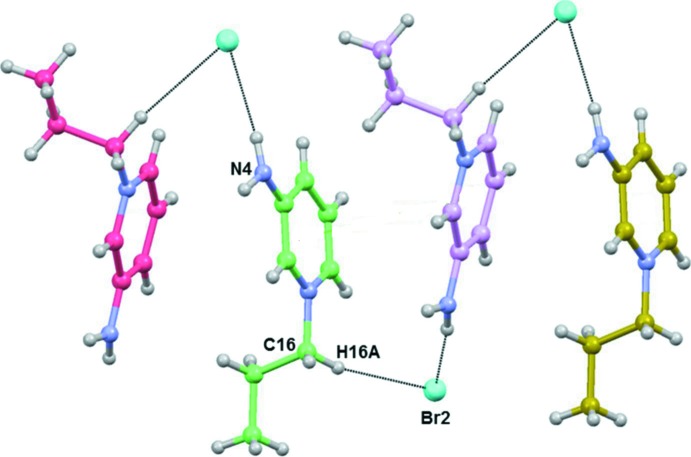
Part of the crystal structure of the title salt, showing the formation of a hydrogen-bonded chain that runs parallel to the *a* axis (see Table 1 ▶ for details; only the inter­acting atoms are labelled).

The crystal structure of the title salt, is stabilized by a network of inter­molecular N—H⋯Br and C—H⋯Br hydrogen bonds (Table 1 ▶ and Fig. 3 ▶). Anion Br2 is involved in five hydrogen bonds as an acceptor while anion Br1 is involved in only two hydrogen bonds. The dimerization of cation *A* mediates through two bromide anions with the aid of two N—H⋯Br and C—H⋯Br hydrogen bonds. As shown in Fig. 4 ▶, these inter­actions generate an 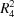
(12) loop. Atom C16 (*via* H16*A*) forms a C—H⋯Br^i^ hydrogen bond with bromide anion Br2 [symmetry code: (i) *x* + 1, *y*, *z*]. The same Br2 anion acts as an acceptor for an N—H⋯Br hydrogen bond with atom N4 of cation *B*. These inter­actions form a chain which runs parallel to the *a* axis (Fig. 5 ▶).

## Database survey   

A search of the Cambridge Structural Database (Version 5.35, last update May 2014; Groom & Allen, 2014 ▶) for 4-amino­pyridinium halide salts gave nine hits, while a search for 3-amino­pyridinium salts yielded eight hits. They all have different substituents at the pyridine ring N position, and include for example, 2-(3-amino­pyridinium-1-yl)propano­ate hydro­bromide hemihydrate (CCDC refcode: IVAWUY; Kowalczyk *et al.*, 2011 ▶), 2-(3-amino­pyridinium-1-yl)-3-carb­oxy­propano­ate monohydrate (CCDC refcode: LAQGAN; Millán Corrales *et al.*, 2012 ▶), 3-amino-1-(carb­oxy­meth­yl)pyridinium chloride (CCDC refcode: PABTIX; Kowalczyk *et al.*, 2010 ▶) and 3-Amino-1-(4-nitro­benz­yl)pyridinium bromide (CCDC refcode: XEBFUG; Sundar *et al.*, 2006 ▶). The mean planes of the substituent groups at the ring N atom make dihedral angles of *ca* 80.3° with the 3-amino­pyridinium ring in IVAWUY and *ca* 86.6° in PABTIX. In LAQGAN, the propano­ate moiety is inclined at an angle of *ca* 86.6°, and the carb­oxy moiety by *ca* 68.4°, with respect to the 3-amino­pyridinium ring. In XEBFUG, the 4-nitro­benzyl ring makes a dihedral angle of *ca* 88.7 ° with the 3-amino­pyridinium ring.

## Synthesis and crystallization   

The title salt was prepared by dissolving 3-amino­pyridine (0.94 g, 10 m*M*) in dried acetone (20 ml) and adding *n*-propyl bromide (1.48g, 12 m*M*). The reaction mixture was stirred at room temperature for 8 h. The title salt precipitated as a white solid, which was filtered and washed with cold acetone and dried in vacuum to afford the stable salt. It was recrystallized from an aqueous ethanol solution giving colourless prismatic crystals.

## Refinement   

The details of crystal data, data collection and structure refinement are summarized in Table 2 ▶. The N-bound H atoms were located in a difference Fourier map and freely refined. In the final cycles of refinement, the H atoms bound to atom N2 were refined with *U*_iso_(H) = 1.1*U*_eq_(N). The C-bound H atoms were included in calculated positions and treated as riding atoms: C—H = 0.93–0.97 Å with *U*_iso_(H) = 1.5*U*_eq_(C) for methyl H atoms and = 1.2*U*_eq_(C) for other H atoms.

## Supplementary Material

Crystal structure: contains datablock(s) I. DOI: 10.1107/S1600536814025665/su5025sup1.cif

Structure factors: contains datablock(s) I. DOI: 10.1107/S1600536814025665/su5025Isup2.hkl

Supporting information file. DOI: 10.1107/S1600536814025665/su5025Isup3.cml

CCDC reference: 1035511

Additional supporting information:  crystallographic information; 3D view; checkCIF report

## Figures and Tables

**Table 1:** Hydrogen-bond geometry (, )

*D*H*A*	*D*H	H*A*	*D* *A*	*D*H*A*
N2H2*A*Br2^i^	0.90(2)	2.47(2)	3.364(3)	177(4)
N2H2*B*Br2^ii^	0.90(2)	2.54(2)	3.419(3)	168(4)
N4H4*A*Br1^iii^	0.83(2)	2.58(2)	3.406(3)	172(3)
N4H4*B*Br2^iv^	0.87(2)	2.57(2)	3.434(3)	171(3)
C6H6*A*Br2	0.97	2.88	3.655(4)	138
C6H6*B*Br1^ii^	0.97	2.84	3.775(4)	163
C16H16*A*Br2^v^	0.97	2.91	3.866(3)	167

Symmetry codes: (i) 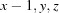; (ii) 
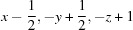
; (iii) 
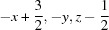
; (iv) 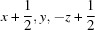; (v) 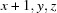.

**Table 2:** Experimental details

Crystal data	
Chemical formula	C_8_H_13_N_2_^+^Br
*M*_r_	217.11
Crystal system, space group	Orthorhombic, *P* *b* *c* *a*
Temperature (K)	296
*a*, *b*, *c* ()	8.2937(1), 17.4137(3), 26.9626(4)
*V* (^3^)	3894.05(10)
*Z*	16
Radiation type	Mo *K*
(mm^1^)	4.17
Crystal size (mm)	0.12 0.10 0.10

Data collection	
Diffractometer	Bruker *SMART* CCD area detector
Absorption correction	Multi-scan (*SADABS*; Bruker, 2008 ▶)
*T*_min_, *T*_max_	0.635, 0.681
No. of measured, independent and observed [*I* > 2(*I*)] reflections	21922, 4491, 2698
*R*_int_	0.043
(sin /)_max_ (^1^)	0.651

Refinement	
*R*[*F* ^2^ > 2(*F* ^2^)], *wR*(*F* ^2^), *S*	0.036, 0.085, 1.00
No. of reflections	4491
No. of parameters	214
No. of restraints	4
H-atom treatment	H atoms treated by a mixture of independent and constrained refinement
_max_, _min_ (e ^3^)	0.44, 0.32

Computer programs: *SMART* and *SAINT* (Bruker, 2008 ▶), *SHELXS2014* and *SHELXL2014* (Sheldrick, 2008 ▶), *PLATON* (Spek, 2009 ▶), *Mercury* (Macrae *et al.*, 2008 ▶) and *PLATON* (Spek, 2009 ▶).

## supplementary crystallographic information

### Crystal data

**Table:**

C_8_H_13_N_2_^+^·Br^−^	*D*_x_ = 1.481 Mg m^−^^3^
*M**_r_* = 217.11	Mo *K*α radiation, λ = 0.71073 Å
Orthorhombic, *P**b**c**a*	Cell parameters from 5144 reflections
*a* = 8.2937 (1) Å	θ = 2.8–23.4°
*b* = 17.4137 (3) Å	µ = 4.17 mm^−^^1^
*c* = 26.9626 (4) Å	*T* = 296 K
*V* = 3894.05 (10) Å^3^	Prismolourec, colourless
*Z* = 16	0.12 × 0.10 × 0.10 mm
*F*(000) = 1760	

### Data collection

**Table:**

Bruker SMART CCD area-detector diffractometer	4491 independent reflections
Radiation source: sealed tube	2698 reflections with *I* > 2σ(*I*)
Graphite monochromator	*R*_int_ = 0.043
phi and ω scans	θ_max_ = 27.6°, θ_min_ = 2.8°
Absorption correction: multi-scan (*SADABS*; Bruker, 2008)	*h* = −10→10
*T*_min_ = 0.635, *T*_max_ = 0.681	*k* = −22→22
21922 measured reflections	*l* = −35→30

### Refinement

**Table:**

Refinement on *F*^2^	Hydrogen site location: mixed
Least-squares matrix: full	H atoms treated by a mixture of independent and constrained refinement
*R*[*F*^2^ > 2σ(*F*^2^)] = 0.036	*w* = 1/[σ^2^(*F*_o_^2^) + (0.0353*P*)^2^ + 1.288*P*] where *P* = (*F*_o_^2^ + 2*F*_c_^2^)/3
*wR*(*F*^2^) = 0.085	(Δ/σ)_max_ < 0.001
*S* = 1.00	Δρ_max_ = 0.44 e Å^−^^3^
4491 reflections	Δρ_min_ = −0.32 e Å^−^^3^
214 parameters	Extinction correction: *SHELXL2014* (Sheldrick, 2008), Fc^*^=kFc[1+0.001xFc^2^λ^3^/sin(2θ)]^-1/4^
4 restraints	Extinction coefficient: 0.00312 (18)

### Special details

**Table:**

Experimental. The minimum and maximum absorption values stated above are those calculated in *SHELXL2014*/6 from the given crystal dimensions. The ratio of minimum to maximum apparent transmission was determined experimentally as 0.639091.
Geometry. All e.s.d.'s (except the e.s.d. in the dihedral angle between two l.s. planes) are estimated using the full covariance matrix. The cell e.s.d.'s are taken into account individually in the estimation of e.s.d.'s in distances, angles and torsion angles; correlations between e.s.d.'s in cell parameters are only used when they are defined by crystal symmetry. An approximate (isotropic) treatment of cell e.s.d.'s is used for estimating e.s.d.'s involving l.s. planes.

### Fractional atomic coordinates and isotropic or equivalent isotropic displacement parameters (Å^2^)

**Table:**

	*x*	*y*	*z*	*U*_iso_*/*U*_eq_	
Br1	0.82809 (4)	0.05269 (2)	0.70989 (2)	0.06468 (14)	
N1	−0.0380 (3)	0.39600 (15)	0.40238 (10)	0.0563 (7)	
N2	−0.3901 (4)	0.3230 (2)	0.46352 (12)	0.0885 (10)	
H2A	−0.472 (3)	0.2941 (19)	0.4526 (13)	0.097*	
H2B	−0.341 (4)	0.305 (2)	0.4909 (10)	0.097*	
C1	−0.1328 (4)	0.36563 (18)	0.43751 (12)	0.0566 (8)	
H1	−0.0897	0.3536	0.4684	0.068*	
C4	−0.2546 (4)	0.4017 (2)	0.34649 (13)	0.0644 (9)	
H4	−0.2950	0.4143	0.3154	0.077*	
C2	−0.2949 (4)	0.35190 (19)	0.42823 (12)	0.0556 (8)	
C7	0.1580 (4)	0.4864 (2)	0.43960 (13)	0.0626 (9)	
H7A	0.0959	0.4875	0.4701	0.075*	
H7B	0.1185	0.5272	0.4183	0.075*	
C3	−0.3540 (4)	0.37094 (18)	0.38170 (12)	0.0601 (9)	
H3	−0.4622	0.3627	0.3744	0.072*	
C8	0.3336 (4)	0.5006 (2)	0.45141 (14)	0.0739 (10)	
H8A	0.3448	0.5494	0.4675	0.111*	
H8B	0.3724	0.4608	0.4730	0.111*	
H8C	0.3951	0.5005	0.4212	0.111*	
C6	0.1340 (4)	0.4116 (2)	0.41464 (13)	0.0653 (9)	
H6A	0.1739	0.3711	0.4360	0.078*	
H6B	0.1969	0.4108	0.3843	0.078*	
C5	−0.0945 (5)	0.4141 (2)	0.35731 (13)	0.0658 (9)	
H5	−0.0260	0.4348	0.3335	0.079*	
Br2	0.29379 (4)	0.21682 (2)	0.42595 (2)	0.06172 (14)	
N3	0.9614 (3)	0.15602 (14)	0.30646 (8)	0.0478 (6)	
N4	0.8705 (4)	0.1118 (2)	0.17923 (11)	0.0678 (8)	
H4A	0.815 (3)	0.0725 (14)	0.1844 (13)	0.064 (11)*	
H4B	0.859 (4)	0.1350 (18)	0.1507 (8)	0.072 (11)*	
C11	0.9112 (3)	0.11840 (17)	0.26620 (10)	0.0455 (7)	
H11	0.8716	0.0687	0.2694	0.055*	
C16	0.9419 (4)	0.1197 (2)	0.35593 (10)	0.0558 (8)	
H16A	1.0258	0.1379	0.3780	0.067*	
H16B	0.9528	0.0644	0.3528	0.067*	
C13	0.9756 (4)	0.22690 (19)	0.21730 (12)	0.0612 (9)	
H13	0.9786	0.2522	0.1869	0.073*	
C15	1.0220 (4)	0.22732 (19)	0.30409 (13)	0.0605 (9)	
H15	1.0591	0.2517	0.3326	0.073*	
C12	0.9171 (3)	0.15187 (18)	0.21987 (10)	0.0477 (7)	
C14	1.0285 (4)	0.26351 (19)	0.25917 (14)	0.0679 (9)	
H14	1.0690	0.3132	0.2570	0.081*	
C18	0.7575 (5)	0.1097 (3)	0.42914 (14)	0.1018 (15)	
H18A	0.6522	0.1233	0.4411	0.153*	
H18B	0.7691	0.0549	0.4294	0.153*	
H18C	0.8379	0.1323	0.4502	0.153*	
C17	0.7780 (4)	0.1388 (3)	0.37761 (13)	0.0812 (12)	
H17A	0.7637	0.1940	0.3775	0.097*	
H17B	0.6949	0.1166	0.3567	0.097*	

### Atomic displacement parameters (Å^2^)

**Table:**

	*U*^11^	*U*^22^	*U*^33^	*U*^12^	*U*^13^	*U*^23^
Br1	0.0623 (2)	0.0678 (3)	0.0640 (2)	−0.01030 (16)	0.00528 (16)	−0.00851 (18)
N1	0.0529 (16)	0.0501 (16)	0.0659 (18)	−0.0015 (13)	0.0095 (14)	−0.0127 (14)
N2	0.083 (2)	0.111 (3)	0.072 (2)	−0.037 (2)	−0.0128 (18)	0.011 (2)
C1	0.061 (2)	0.053 (2)	0.056 (2)	−0.0037 (16)	−0.0051 (16)	−0.0058 (16)
C4	0.070 (2)	0.061 (2)	0.062 (2)	−0.0034 (19)	0.0026 (19)	−0.0106 (18)
C2	0.056 (2)	0.053 (2)	0.058 (2)	−0.0156 (15)	0.0041 (16)	−0.0062 (17)
C7	0.057 (2)	0.063 (2)	0.068 (2)	0.0004 (17)	0.0003 (16)	−0.0092 (19)
C3	0.056 (2)	0.061 (2)	0.063 (2)	−0.0030 (17)	−0.0124 (17)	−0.0115 (18)
C8	0.059 (2)	0.079 (3)	0.084 (3)	−0.0115 (19)	−0.0043 (18)	0.002 (2)
C6	0.0474 (19)	0.064 (2)	0.085 (2)	−0.0005 (17)	0.0056 (17)	−0.008 (2)
C5	0.080 (3)	0.060 (2)	0.057 (2)	−0.0028 (19)	0.0154 (19)	−0.0059 (18)
Br2	0.0741 (3)	0.0604 (2)	0.0506 (2)	−0.01163 (16)	0.00011 (15)	−0.00427 (16)
N3	0.0482 (14)	0.0465 (16)	0.0486 (15)	0.0023 (12)	−0.0029 (11)	0.0027 (12)
N4	0.087 (2)	0.072 (2)	0.0449 (18)	−0.0176 (18)	−0.0075 (16)	0.0096 (17)
C11	0.0466 (17)	0.0398 (17)	0.0500 (17)	−0.0013 (13)	−0.0030 (14)	0.0008 (15)
C16	0.057 (2)	0.063 (2)	0.0474 (17)	−0.0012 (16)	−0.0069 (15)	0.0026 (16)
C13	0.069 (2)	0.056 (2)	0.058 (2)	−0.0019 (17)	0.0035 (17)	0.0141 (18)
C15	0.068 (2)	0.049 (2)	0.064 (2)	−0.0054 (17)	−0.0065 (17)	−0.0074 (17)
C12	0.0483 (17)	0.0518 (19)	0.0430 (18)	−0.0003 (14)	−0.0023 (14)	0.0074 (15)
C14	0.079 (2)	0.045 (2)	0.080 (3)	−0.0076 (17)	−0.001 (2)	0.0045 (19)
C18	0.086 (3)	0.141 (4)	0.078 (3)	0.011 (3)	0.015 (2)	0.017 (3)
C17	0.066 (2)	0.115 (3)	0.063 (2)	0.003 (2)	0.0002 (18)	0.022 (2)

### Geometric parameters (Å, º)

**Table:**

N1—C5	1.340 (4)	N3—C11	1.334 (3)
N1—C1	1.340 (4)	N3—C15	1.341 (4)
N1—C6	1.489 (4)	N3—C16	1.485 (3)
N2—C2	1.336 (4)	N4—C12	1.356 (4)
N2—H2A	0.896 (18)	N4—H4A	0.834 (18)
N2—H2B	0.897 (18)	N4—H4B	0.874 (18)
C1—C2	1.388 (4)	C11—C12	1.379 (4)
C1—H1	0.9300	C11—H11	0.9300
C4—C3	1.366 (4)	C16—C17	1.517 (4)
C4—C5	1.377 (5)	C16—H16A	0.9700
C4—H4	0.9300	C16—H16B	0.9700
C2—C3	1.387 (4)	C13—C14	1.369 (4)
C7—C6	1.479 (4)	C13—C12	1.395 (4)
C7—C8	1.511 (4)	C13—H13	0.9300
C7—H7A	0.9700	C15—C14	1.366 (4)
C7—H7B	0.9700	C15—H15	0.9300
C3—H3	0.9300	C14—H14	0.9300
C8—H8A	0.9600	C18—C17	1.488 (5)
C8—H8B	0.9600	C18—H18A	0.9600
C8—H8C	0.9600	C18—H18B	0.9600
C6—H6A	0.9700	C18—H18C	0.9600
C6—H6B	0.9700	C17—H17A	0.9700
C5—H5	0.9300	C17—H17B	0.9700
			
C5—N1—C1	121.9 (3)	C11—N3—C15	122.2 (3)
C5—N1—C6	119.6 (3)	C11—N3—C16	119.2 (3)
C1—N1—C6	118.5 (3)	C15—N3—C16	118.6 (3)
C2—N2—H2A	115 (3)	C12—N4—H4A	116 (2)
C2—N2—H2B	117 (2)	C12—N4—H4B	120 (2)
H2A—N2—H2B	115 (4)	H4A—N4—H4B	118 (3)
N1—C1—C2	120.6 (3)	N3—C11—C12	121.2 (3)
N1—C1—H1	119.7	N3—C11—H11	119.4
C2—C1—H1	119.7	C12—C11—H11	119.4
C3—C4—C5	119.7 (3)	N3—C16—C17	110.5 (2)
C3—C4—H4	120.1	N3—C16—H16A	109.6
C5—C4—H4	120.1	C17—C16—H16A	109.6
N2—C2—C3	121.7 (3)	N3—C16—H16B	109.6
N2—C2—C1	120.6 (3)	C17—C16—H16B	109.6
C3—C2—C1	117.7 (3)	H16A—C16—H16B	108.1
C6—C7—C8	111.6 (3)	C14—C13—C12	120.4 (3)
C6—C7—H7A	109.3	C14—C13—H13	119.8
C8—C7—H7A	109.3	C12—C13—H13	119.8
C6—C7—H7B	109.3	N3—C15—C14	119.0 (3)
C8—C7—H7B	109.3	N3—C15—H15	120.5
H7A—C7—H7B	108.0	C14—C15—H15	120.5
C4—C3—C2	120.6 (3)	N4—C12—C11	120.3 (3)
C4—C3—H3	119.7	N4—C12—C13	122.8 (3)
C2—C3—H3	119.7	C11—C12—C13	116.9 (3)
C7—C8—H8A	109.5	C15—C14—C13	120.2 (3)
C7—C8—H8B	109.5	C15—C14—H14	119.9
H8A—C8—H8B	109.5	C13—C14—H14	119.9
C7—C8—H8C	109.5	C17—C18—H18A	109.5
H8A—C8—H8C	109.5	C17—C18—H18B	109.5
H8B—C8—H8C	109.5	H18A—C18—H18B	109.5
C7—C6—N1	113.0 (3)	C17—C18—H18C	109.5
C7—C6—H6A	109.0	H18A—C18—H18C	109.5
N1—C6—H6A	109.0	H18B—C18—H18C	109.5
C7—C6—H6B	109.0	C18—C17—C16	112.8 (3)
N1—C6—H6B	109.0	C18—C17—H17A	109.0
H6A—C6—H6B	107.8	C16—C17—H17A	109.0
N1—C5—C4	119.6 (3)	C18—C17—H17B	109.0
N1—C5—H5	120.2	C16—C17—H17B	109.0
C4—C5—H5	120.2	H17A—C17—H17B	107.8
			
C5—N1—C1—C2	−0.1 (5)	C15—N3—C11—C12	−1.1 (4)
C6—N1—C1—C2	178.3 (3)	C16—N3—C11—C12	175.7 (3)
N1—C1—C2—N2	−178.7 (3)	C11—N3—C16—C17	−88.0 (3)
N1—C1—C2—C3	−0.2 (5)	C15—N3—C16—C17	88.9 (3)
C5—C4—C3—C2	−0.1 (5)	C11—N3—C15—C14	1.9 (5)
N2—C2—C3—C4	178.8 (3)	C16—N3—C15—C14	−174.9 (3)
C1—C2—C3—C4	0.3 (5)	N3—C11—C12—N4	177.9 (3)
C8—C7—C6—N1	−179.9 (3)	N3—C11—C12—C13	−0.9 (4)
C5—N1—C6—C7	94.3 (4)	C14—C13—C12—N4	−176.8 (3)
C1—N1—C6—C7	−84.2 (4)	C14—C13—C12—C11	1.9 (4)
C1—N1—C5—C4	0.4 (5)	N3—C15—C14—C13	−0.8 (5)
C6—N1—C5—C4	−178.0 (3)	C12—C13—C14—C15	−1.1 (5)
C3—C4—C5—N1	−0.3 (5)	N3—C16—C17—C18	−174.8 (3)

### Hydrogen-bond geometry (Å, º)

**Table:**

*D*—H···*A*	*D*—H	H···*A*	*D*···*A*	*D*—H···*A*
N2—H2*A*···Br2^i^	0.90 (2)	2.47 (2)	3.364 (3)	177 (4)
N2—H2*B*···Br2^ii^	0.90 (2)	2.54 (2)	3.419 (3)	168 (4)
N4—H4*A*···Br1^iii^	0.83 (2)	2.58 (2)	3.406 (3)	172 (3)
N4—H4*B*···Br2^iv^	0.87 (2)	2.57 (2)	3.434 (3)	171 (3)
C6—H6*A*···Br2	0.97	2.88	3.655 (4)	138
C6—H6*B*···Br1^ii^	0.97	2.84	3.775 (4)	163
C16—H16*A*···Br2^v^	0.97	2.91	3.866 (3)	167

Symmetry codes: (i) *x*−1, *y*, *z*; (ii) *x*−1/2, −*y*+1/2, −*z*+1; (iii) −*x*+3/2, −*y*, *z*−1/2; (iv) *x*+1/2, *y*, −*z*+1/2; (v) *x*+1, *y*, *z*.
